# Supplementary material for: Are carbon emissions trading and green financial instruments synergistic? -Comprehensive quantitative research based on content analysis
Source: PLoS One. 2024 Mar 7;19(3):e0298601. doi: 10.1371/journal.pone.0298601 (PMC10919601; doi:10.1371/journal.pone.0298601)
Supplement: S1 Data — (ZIP) [file pone.0298601.s001.zip › Data/地级市Z_F工作报告.zip!/░▓╗╒/╬▀║■╩╨/└┤╫╘╣1⁄2╓┌║┼í╛┬φ┐╦╩2╛▌═°í┐_2018.docx]

各位代表：

现在，我代表市人民政府，向大会报告政府工作，请予审议，并请市政协各位委员提出意见。

一、2017年和本届政府工作回顾

2017年，全市人民在中共芜湖市委的坚强领导下，深入贯彻习近平总书记视察安徽重要讲话精神，全面落实中央和省委、省政府决策部署，践行新发展理念，坚持稳中求进工作总基调，实施五大发展行动计划，经济社会保持健康较快发展势头，较好完成了市十五届人大七次会议确定的主要目标任务。

全市生产总值3066亿元（初步核算数，下同），比上年增长8.9%；财政收入558亿元，增长9%；固定资产投资3342亿元，增长11.2%；社会消费品零售总额931亿元，增长12.4%；进出口额63.8亿美元，增长12.3%。居民人均可支配收入28622元，增长9.4%；城镇新增就业7.7万人、登记失业率3.4%；单位生产总值能耗下降7.7%；生态环境质量稳中向好。

一年来，我们主要做了以下几个方面的工作。

（一）抓产业、扶实体，稳住经济增长。

强服务促发展。突出抓好企业降本减负，出台促进经济平稳健康发展的32条意见、扶持产业发展"1+5+6"政策体系。深化“一企一组一策”帮扶行动，实施“四送一服”双千工程，兑付支持企业发展奖补资金36亿元，减轻企业税费负担超百亿元。突出抓好融资服务，新增众源新材上市首发、15家企业新三板挂牌，全市直接融资380亿元。年末金融机构存贷款余额分别达3255亿元、3211亿元，分别比年初净增339亿元、359亿元。突出抓好创新资源集聚，出台加快推进芜湖国家自主创新示范区建设的若干政策，新引进高层次科技人才团队45个。中电科钻石飞机公司获批国家地方联合工程研究中心，中国电信集团（芜湖）云计算中心建成，设立哈尔滨工业大学、江苏大学技术转移转化中心和西安电子科技大学芜湖研究院。万人有效发明专利拥有量达26.1件，保持全省第一。新认定高新技术企业63家，高新技术产业增加值增长14.8%。高新技术产业开发区获批国家级“双创”示范基地。

强产业促升级。传统产业加快转型，汽车及零部件、材料、电子电器、电线电缆四个支柱产业增加值增长8.3%。奇瑞获评国家级工业设计中心、省政府质量奖。海螺水泥产销量和效益创历史新高。工业技改投资增长33.8%。32家企业实施兼并重组。聚焦培育战略性新兴产业，产值增长22.4%。机器人产业集聚区获评最佳机器人产业园奖。埃夫特收购2家意大利公司。全国首台全铝车身“小蚂蚁”上市，新能源汽车销售增长90.2%。中电科钻石飞机成为全国首家“国外TC+中国PC”飞机制造企业。现代农机逆势增长，产品出口50多个国家和地区。新一代汽车电子、跨座式单轨及APM车辆等5个工程（专项）获批第二批省重大工程、重大专项，我市入选国家通用航空产业综合示范区。服务业发展提速，重点现代服务业营业收入增长16.2%，新增省级服务业集聚区2家。网上商品零售额增长25%，三只松鼠、凡臣电子被评为国家电商示范企业，易酒批、共生物流入选中国产业互联网企业百强榜。八佰伴生活广场开业。快递业务量突破1亿件。全市接待游客4800万人次，方特门票收入居全省第一。大浦获批国家级水利风景区。成功举办中国国际动漫创意产业交易会、中国技能大赛、技能中国行——走进安徽、中国海归创业大赛、全国机器人专利创新创业大赛。广告产业园区获批国家广告产业园。数字出版园区获评全国优秀园区。现代农业稳步发展，“三品一标”有效认证322个，农产品加工产值增长13.5%。

强投资增后劲。新开工亿元以上项目572个，其中10亿元以上项目52个；在建亿元以上项目1323个，其中10亿元以上项目228个。实际利用外资26.9亿美元。民间投资增长5%。42家企业入选安徽省百强民营企业，居全省第一。

（二）抓统筹、强功能，推进城乡建设。

高起点加强城市规划。修编了《芜湖市城市总体规划》、《江北产业集中区总体规划》。开展了市县“多规合一”试点及城市空间规划、城市景观风貌规划等编制，城市专项规划实现全覆盖。无为撤县设市方案已报国务院。全市结束乡行政体制。

高标准优化城市交通。长江公路二桥、北沿江高速巢无段、庐铜铁路、通江大道一级公路改建、芜合高速二坝互通、沿江高速峨山路互通、弋江路快速化改造米市口立交和马塘立交、芜申运河张镇码头等工程基本建成。火车站西站房、商合杭铁路长江公铁大桥、机场、轨道交通等加快建设。芜黄高速、宁芜高速芜湖东互通改建、皖江第一隧——城南过江隧道、全国首个大盾构基地配套码头等项目开工。我市入选公交都市创建城市。

高水平建设美丽家园。编制了2018～2020年棚户区改造计划，完成棚改2.3万套。在全省率先开展住房租赁试点，超额完成政府投资公租房分配任务，房地产市场保持平稳健康发展。九华南路、桥头公园、滨江公园二期等城市品质提升项目开工。新开工排水防涝项目58个，建成双摆渡等大型排涝泵站。完成29条明渠清淤清障，31条黑臭水体、18个排涝隐患点、21个老旧小区、14个农贸市场整治。启动4个污水处理厂提标改造工程。孙村镇入选全国特色小镇。

（三）抓改革、促开放，增强发展活力。

加快推进政府职能转变。组建市投资促进局。建成市县两级行政权力中介服务清单。建成全国领先的政府大数据库，市级5164项、各县区18822项行政权力和公共服务事项全部实现网上运行。易政网投入试运行。深化商事制度改革，发出全省首张“多证合一”营业执照，推进企业登记注册全程电子化，在全省率先推行企业登记 “容缺受理”制度。各类市场主体达25.2万户，增长17.5%。

加快推进重点领域改革。完成城管执法体制改革。完善政府采购制度，公共资源交易市县一体化改革全面完成，“徽采商城”上线运行。新增政府债券20亿元，发行置换债券129亿元，年节约资金成本6亿元。创新开展中小企业信用贷试点。事业单位分类改革扎实推进。实行市属国企负责人薪酬制度改革，加强国资国企重大事项管理。实施了中小学教师职称评审改革。

加快推进开放平台建设。港口货物吞吐量1.3亿吨，集装箱量70万标箱。肉类进境指定口岸获批。综合保税区进出口额11.7亿美元。复制推广19项自贸区经验。成立了中德人工智能研究院。实际对外投资3.5亿美元。

（四）抓生态、优环境，推进绿色发展。

在节能减排上下功夫。落实能耗和总量“双控”目标，单位工业增加值能耗下降9.1%。加强再生能源利用，牛埠50兆瓦光伏电站、石涧50兆瓦风电场等建成投产。实施重点减排项目16个。完成大气污染整治项目117个，淘汰、改造燃煤小锅炉12台。

在增绿增效上下功夫。完成造林15万亩、森林长廊166公里，新增绿地324万平方米、绿道61公里。开展9家矿山治理。划定全域永久基本农田332万亩，整理复垦开发补充耕地1.3万亩。盘活工业用地5381亩，收回闲置低效土地4481亩。全面推行河长制，全国水生态文明城市建设试点通过评估。

在中央、省环保督查反馈问题整改上下功夫。坚持为国护江、为民治江、为城建江，解决了二水厂水源地中石化码头搬迁、箱子拐长江岸线整治等一批长期想解决而没有解决的环境难题。完成长江干流岸线整治项目191个，释放长江干流岸线27公里、滩涂陆域面积244万平方米。

（五）抓民生、兜底线，共享发展成果。

脱贫攻坚扎实推进。始终把脱贫攻坚作为重大政治任务和最大民生工程，开展“重精准、补短板、促攻坚”专项行动，推动政策落实，安排专项扶贫资金3亿元，实现贫困村产业扶贫项目、贫困户帮扶责任人全覆盖，年度减贫任务顺利完成。

文明创建不断深入。开展“贴近群众六走进”行动，一批群众关注的热点难点问题得到解决。启动“清洁城乡、美化家园”环境整治和农村垃圾、污水、厕所“三大革命”，出台了加强市区公厕建设管理、农贸市场整治提升工作方案，开展市容市貌提升专项行动，整治餐饮油烟、广告标牌、建筑扬尘、积存垃圾等。我市蝉联全国文明城市，新创8个全国文明单位（村镇、校园）。

民生保障体系日益健全。33项民生工程完成投入108.5亿元。城镇职工五项保险参保280万人次，企业基本养老金月人均增资125元。完善职工医保住院医疗费用结算办法，完成跨省异地就医联网直接结算，15家医疗机构纳入全国结算系统。完成就业技能培训、岗位技能提升培训2.8万人。出台支持技工强市建设政策。发放低保金、医疗救助和临时救助金5.9亿元。开展农村留守儿童关爱保护工作。发放人才购房安家补助3亿元。新增住房公积金缴存人数5.3万，发放贷款26.7亿元。加强了电梯安全监管。

社会事业蓬勃发展。新成立2个教育集团，14个名校长工作室，优质学校办分校（分园）3个。农村中小学校舍维修改造项目开工152个，第二十七中学东方龙城校区、第二十八中学建成开学。新设立芜湖京师职业学校，安徽工程大学国际工程师学院开工。成立17个医联体、8个县域医共体，提升基层卫生服务能力，推进分级诊疗和家庭医生签约服务。芜湖县、繁昌县获评国家卫生县城。萧瀚美术馆建成开放，举办了刘开渠奖国际雕塑大展、皖江八市群艺大赛、市第十三届运动会暨首届市民运动会和首届残疾人运动会。高质量完成第三次全国农业普查。市烈士陵园实施整体改造。开发运行“城市令”“今日芜湖”和“全民社管”平台。深化“平安芜湖”建设，我市成为省食品药品安全城市，安全生产形势向好，社会保持和谐稳定。

去年，政府系统扎实推进“两学一做”学习教育常态化制度化，开展了“讲政治、重规矩、作表率”专题警示教育。全面实施“七五”普法规划，加强行政复议和应诉工作，提高依法行政水平。办理人大建议144件、政协提案266件。深入开展“亲切服务”，市政务服务中心获批国家级社会管理和公共服务综合标准化试点。12345政府服务直通车整合电梯应急救援等热线。坚持审计全覆盖，完善惩治和预防腐败体系，12388纪检监察网络举报平台、“小微权力”网络监督平台上线运行。查处违反中央八项规定精神典型问题221起，给予党政纪处分245人。

各位代表！

本届政府工作这五年，是全市人民砥砺奋进的五年，是芜湖发展浓墨重彩的五年。芜湖在全省经济总量第二的地位全面巩固，在长三角城市群、中部省份副中心城市和沿江城市中的战略地位明显提升。

五年来，我们始终以质量效益为导向，综合实力进一步提升。地区生产总值由1874亿元增加到3066亿元，年均增长10.3%，增速居全省前列。规模工业主营业务收入超6000亿元，利润总额近300亿元。五年累计完成固定资产投资1.3万亿元，年均增长14.4%。财政收入由337亿元增加到558亿元，年均增长10.6%。中国驰名商标由14件增加到37件。

五年来，我们始终以科技创新为引领，产业结构进一步优化。三次产业比重由6.3∶65.9∶27.8调整为4.2∶56∶39.8。支柱产业对规模工业增长贡献率保持在65%，战略性新兴产业产值占规模工业比重由17%提升到25.9%。机器人产业在国内率先形成全产业链集聚发展态势。服务业集聚区建设在全省领先。网上零售额跃居全省首位，快递业务量稳居中部非省会城市第一。获批国家自主创新示范区，省级以上研发机构306家，高新技术企业520家。万人有效发明专利拥有量、高新技术产业增加值比重稳居全省第一。

五年来，我们始终以改革开放为路径，发展活力进一步释放。综合监管分类执法、棚改货币化安置、住房金融服务、信用体系建设、“互联网+政务服务”、工业用地市场化配置、农村公共建设管理体制等改革走在全省全国前列。系统推进全面创新改革试验取得积极成效。深化“放管服”改革，在全省率先建成市县乡三级政府权责清单、公共服务清单体系，率先公布涉企收费清单、企业投资准入负面清单。国务院通报表扬的落实有关重大政策措施真抓实干成效明显的9项工作中，芜湖有4项跻身其中。上市公司由11家增加到17家，累计实际利用外资111.7亿美元、对外投资17.2亿美元。经济技术开发区综合发展水平进入全国20强，综合保税区跨入全国海关特殊监管区域50强。芜湖港成为安徽省首个亿吨大港，享受启运港退税政策。

五年来，我们始终以功能品质为核心，城乡环境进一步改善。我市被列入国家长三角城市群发展规划大城市。市区面积由1065平方公里扩大到1491平方公里，建成区面积达175平方公里，城镇化率由58%上升到64%。累计完成棚户区改造11万套。青弋江分洪道工程建成受益。京福、宁安高铁通车，实现县县通高速公路，公路网密度居全省第一。新建扩建7座公园，新增绿地2017万平方米，森林覆盖率由17.9%上升到23.2%。

五年来，我们始终以惠民利民为宗旨，群众获得感进一步增强。累计脱贫11.5万人，71个贫困村全部出列。滚动实施55项民生工程，累计投入461亿元。完成453所义务教育学校标准化建设，全市实现义务教育均衡发展目标。学前三年毛入园率由84%提高到92%，高中阶段毛入学率由89%提高到98%。引进北师大、华师大等优质教育资源，教育信息化全国领先。累计新增就业43.3万人。医疗服务体系更加完善，获批公立医院综合改革、基层综合医改、精神卫生综合示范、医养结合等国家级试点，在全省率先实施大病医疗补充保险，在全国率先实施市县两级公立医院药品零加成销售改革。成立全国首家地方住房金融公司，在全国率先实施进城务工农民缴存使用住房公积金制度。农村金融服务室实现全覆盖。

五年来，我们始终以形象品牌为支撑，城市影响力进一步扩大。市博物馆、规划馆、滨江文化艺术中心建成，93个公共文化场馆全部免费开放，雨耕山、大砻坊再现生机。方特旅游区成功创建国家5A级景区，全市游客接待量年均增长23.4%。中国动漫会、中国（芜湖）科博会、中国机器人产业推进大会、刘开渠奖国际雕塑大展等会展反响热烈。成功创建全国文明城市、电子商务示范城市、信息消费示范城市、国土资源节约集约模范市、信息惠民试点城市、智慧城市试点城市、文化消费试点城市，蝉联全国双拥模范城。

各位代表！

这些成绩的取得，是中央、省委省政府和市委正确领导的结果，是市人大、市政协加强监督和支持的结果，是全市人民共同拼搏的结果。每一个芜湖市民，都是这段辉煌历程的参与者和见证者。在这里，我代表芜湖市人民政府，向全市人民，向全体人大代表和政协委员，向各民主党派、各人民团体和各界人士，向上级部门、兄弟市和驻芜部队、武警官兵，向关心支持芜湖发展的海内外友好人士，表示崇高的敬意和诚挚的感谢!

我们也清醒认识到，芜湖发展中的问题和挑战。主要有：发展不平衡不充分，发展质量和效益还不高，创新要素集聚能力不够强，高层次人才紧缺，资源环境约束趋紧；防范化解重大风险、精准脱贫、污染防治的任务艰巨，安全生产、社会治理存在薄弱环节，基础设施、公共服务有待完善。少数政府部门和工作人员不作为、慢作为、不敢担当现象较为突出，廉政风险依然存在。我们一定正视问题，攻坚克难，全力做好工作，不负人民重托。

二、高质量全面建成小康社会，奋力打造现代化创新之城

未来一个时期，世界经济政治格局仍在深度调整，发展面临的困难仍然较多。我国已进入“两个一百年”奋斗目标的历史交汇期，我们既要全面建成小康社会，又要乘势而上开启全面建设社会主义现代化国家的新征程。在百舸争流的大潮中，市政府必须把思想和行动统一到中央决策部署上来，坚定信心，抢抓机遇，加快质量变革、效率变革、动力变革，推动转型发展、升级发展、特色发展，实现芜湖经济由高速增长向高质量发展转变。

今后五年政府工作总体思路是：全面贯彻党的十九大精神，以习近平新时代中国特色社会主义思想为指导，落实省委、省政府和市委部署，坚持稳中求进工作总基调，坚持新发展理念，以供给侧结构性改革为主线，以五大发展行动计划为总抓手，聚焦建设现代化科教人才体系、现代化产业体系、现代化城乡体系、现代化治理体系，高质量全面建成小康社会，奋力打造现代化创新之城。

认真落实省委、省政府“当好皖江崛起排头兵、五大发展先行者”的要求，今后五年经济社会发展的主要目标是：

综合实力再上新台阶。完成“十三五”规划和决胜全面建成小康社会目标任务，争取“十四五”时期良好开局。地区生产总值突破5000亿元，第三产业比重达55%。创新动能进一步增强，打造长江经济带具有重要影响力的产业创新中心。

城乡建设再现新风貌。拥江发展有序推进，城乡公共资源配置更为均衡，立体交通体系初步建立，“山水相间、精致繁华”的城市风貌特色进一步彰显，城市功能、品质和能级进一步提升。

生态质量再有新提高。主要污染物排放量明显下降，空气、水、土壤污染等影响人民群众健康的突出环境问题有效解决，生态文明制度体系基本形成，芜湖的天更蓝、地更绿、水更清。

开放发展再谱新篇章。高标准大通道、高水平大平台、高效率大通关基本实现，对外贸易、利用外资明显提高，率先建成要素自由流动、资源高效配置、市场深度融合的开放型经济体系。

民生福祉再获新改善。现行标准下农村贫困人口实现脱贫，在幼有所育、学有所教、劳有所得、病有所医、老有所养、住有所居、弱有所扶上不断取得新进展，芜湖人民获得感、幸福感、安全感更加充实。

实现上述目标，要着重把握好以下六个方面：

一是更加注重政治方向，彰显文明行政的正气。坚持党的领导，牢固树立政治意识、大局意识、核心意识、看齐意识，切实增强道路自信、理论自信、制度自信、文化自信，坚决维护党中央权威和集中统一领导，始终同以习近平同志为核心的党中央保持高度一致。坚持执政为民，坚守人民立场、人民中心、人民情怀，抓住老百姓最急最忧最愁的问题，了解群众的操心事、烦心事，把人民对美好生活的向往作为奋斗目标，把人民满意作为政府工作标准。坚持依法行政，建设法治政府，强化法治思维和法治理念，完善公众参与、专家论证、风险评估、合法性审查和集体讨论决定的决策程序，严格规范公正文明执法，织密扎牢制度的笼子，确保权力在阳光下运行，增强政府公信力和执行力。

二是更加注重创新驱动，彰显拥江发展的雄气。坚定实施科教兴市战略、人才强市战略、创新驱动发展战略，持之以恒推进创新型城市、人才特区、创业特区建设，打造中部一流、全国领先的人才高地。深化供给侧结构性改革，加快建设实体经济、科技创新、现代金融、人力资源协同发展的产业体系。系统推进全面创新改革试验，推进合芜蚌国家自主创新示范区建设，形成3~5个国家级重大公共研发平台，引进一大批创新创业团队、领军人才和高端人才。完善扶持产业发展政策体系。加快知识产权强市建设，培育高价值专利和知识产权密集型产业。实施战略性新兴产业“三重一创”工程，鼓励企业实施技术改造，支持奇瑞、海螺进军世界500强。建设全国重要的制造强市、质量强市，区域金融中心、现代物流中心和信息消费服务中心，打造“欢乐芜湖”。通过“区、港、园、桥、隧”联动，形成一条大江育两岸、产城对拥望比邻的新格局。

三是更加注重统筹协调，彰显精致繁华的秀气。坚定实施乡村振兴战略、区域协调发展战略、军民融合发展战略，坚持走内涵式、集约化城市发展之路，在城市规划建设管理上像绣花一样精细，努力在功能品质上与一二线城市等高对接，推动城乡融合发展。推进全国性综合交通枢纽建设，建成民用机场、商合杭高铁、皖赣铁路新建双线、芜合高速扩建、芜黄高速、沪汉高速无为段、芜申运河、芜湖闸、城南过江隧道、城市轨道交通、城区快速路网体系等工程，加快芜宣高速、马芜高速等改造建设，提高国省干道等级。建设智慧城市、公交都市。推进县城建设和美丽乡村环境整治。深化农村土地制度和集体产权制度改革，建设农业绿色精品强市。

四是更加注重生态环境，彰显半城山水的灵气。坚定实施可持续发展战略，践行绿水青山就是金山银山的理念，健全绿色低碳循环发展的经济体系，加强生态系统保护，倡导简约适度、绿色低碳的生活方式。落实主体功能区规划，统筹山水林田湖草系统治理，保护城市肌理，整改突出环境问题。实施植树造林、园林绿化工程。加强岸线资源监管。实施大气、水、土壤污染防治行动计划，坚决打赢污染防治攻坚战。开展节能降碳，控制煤炭消费总量。完善生态环境管理制度，规范自然资源开发利用，坚决查处破坏生态环境行为。争创国家环保模范城市、生态园林城市、森林城市，打造生态文明建设芜湖样板。

五是更加注重秉持开放，彰显中西交融的底气。深化重点领域和关键环节改革，完善市场经济、城市管理、社会治理体制机制。传承芜湖的开放基因，坚持引进来和走出去并重、引资和引才并重、对内开放和对外开放并重，积极参与“一带一路”建设，推动贸易和投资自由化便利化，申报创建自贸试验区，打造内陆开放新高地。拓展对外贸易，培育贸易新业态新模式，加强汽车、电器、农机等出口基地建设，推动外贸向“优质优价、优进优出”转变。推进综合保税区扩区升级，建设保税港区、空港保税物流园区，推进“区港空”一体化发展。深化芜湖港与上海港战略合作。推进开发区转型升级。落实长三角城市群发展规划，深度融入区域分工合作，形成宽领域融合发展新格局。推动文化繁荣兴盛，实施文化惠民工程，加大文化基础设施投入，完善公共文化服务体系，发展特色文化产业，建设文化强市。

六是更加注重普惠共享，彰显美好生活的福气。坚持人民主体地位，把老百姓的安危冷暖时刻放在心上，把为人民造福的事情真正办好办实。优先发展教育事业，打造教育名城，各级各类教育发展水平进入全国同类城市先进行列。提高就业质量和居民收入水平，实施积极就业政策，鼓励勤劳守法致富。加强社会保障体系建设，全面实施全民参保计划。推进棚户区改造，加快建立多主体供给、多渠道保障、租购并举的住房制度。坚决打赢脱贫攻坚战，确保2020年现行标准下所有贫困人口全部脱贫。实施健康芜湖规划，深化医药卫生体制改革，健全城乡医疗卫生服务体系，打造皖江医疗中心。落实生育政策，积极应对人口老龄化。牢固树立安全发展理念，压实安全生产责任。坚决打赢防范化解重大风险攻坚战，依法打击各类违法犯罪活动。健全社区治理体系，发挥社会组织作用，推进社会服务管理信息化，打造共建共治共享的社会治理格局。争创国家卫生城市、食品安全示范城市、社会信用体系建设示范城市。

三、2018年主要工作

2018年是贯彻党的十九大精神的开局之年，是改革开放40周年，是决胜全面建成小康社会、实施“十三五”规划承上启下的关键一年。经济社会发展主要预期目标是：地区生产总值增长8.5%，财政收入增长8%，居民人均可支配收入增长8.5%，城镇新增就业6.5万人，城镇登记失业率、调查失业率分别控制在4.5%和5%以内，节能减排完成省政府下达任务。

重点抓好以下工作：

（一）聚焦质量效益，构建创新体系。

打造产业创新中心。统筹推进“全创改”“合芜蚌”示范区建设。深化人才发展体制机制改革，深入实施“3111”“百千万”人才工程，面向全球招引拔尖人才、领军人才、高端人才和创新创业团队，加快培养软件工程师、机电工程师等高素质技能人才。动态支持首批37个高端研发创新平台建设，突出关键共性技术、前沿引领技术、现代工程技术、颠覆性技术创新，为产业发展提供有力支撑。坚持企业创新主体地位，支持企业提升研发能力，实施重大科技项目。加强与在芜高校合作，吸引国内外创新型企业、知名科研机构、大院大所来芜合作，共建高水平研发机构。鼓励龙头企业通过并购、合资、参股等方式，建立海外研发机构。全面下放高校和科研院所科技成果使用权、处置权和收益权，加快皖江技术转移转化中心、皖江知识产权运营交易中心和科技企业孵化器建设，推动科技成果产业化。提升公共技术服务水平，加快国家工业机器人产品质量监督检验中心（安徽）建设。强化知识产权创造运用保护，推进国家知识产权质押融资试点。

塑造新产业发展体系。坚持有限目标、国内领跑、国际争先，加快战略性新兴产业“三重一创”建设, 扩大机器人及智能装备、新能源汽车、现代农机、通用航空产业规模实力，培育微电子、新型显示、轨道交通装备、新材料、节能环保装备、产业互联网及共享经济等产业。支持南陵打造全国快递科技创新试验基地。实施下一代智能工业机器人、中小型航空发动机、新能源汽车动力电池和电控系统等市级重大新兴产业工程和专项。

改造提升传统优势产业。推动制造业与大数据、物联网、工业设计融合，培育智能工厂、数字化车间。完成工业技改投资750亿元，鼓励企业引进先进技术和装备，攻克制约产业升级难题；支持中小企业走专业化、精细化、特色化、新型化发展路子，培育一批行业“单打冠军”“隐形冠军”。鼓励企业兼并重组，妥善处置“僵尸企业”。落实稳增长政策措施。支持奇瑞、海螺等龙头企业发展，提升电线电缆科技水平，引领带动支柱产业迈向中高端。推动军民融合发展，支持军民融合产业基地建设。

营造现代服务业集聚发展态势。培育金融、现代物流、电商、文化旅游、共享经济、现代供应链、人力资本服务等重点现代服务业，新增1~2家省级服务业集聚区。建设朱家桥、三山、裕溪口等物流园区，加快向现代化综合物流型港口转型。推动互联网、大数据、人工智能和实体经济深度融合，加快“老船厂·智慧港”产业园建设，支持三只松鼠、共生物流、易酒批等企业发展。办好科博会、汽车展等会展活动。

（二）聚焦城乡融合，实现协调发展。

经济景气与文明景象等量平衡。以绣花功夫推进精准扶贫、精准脱贫，以扶贫统领农村各项工作，通过政策叠加、资源叠加和各方力量叠加，打造脱贫攻坚九大工程升级版，巩固脱贫成效，提升脱贫质量，确保完成年度脱贫任务。持续开展“贴近群众六走进”行动，整治交通、市容等乱象，推进农村垃圾、污水、厕所“三大革命”，巩固文明城市创建成果。聚焦群众生产生活，完善基础设施、公共服务，补齐民生短板，加强未成年人思想道德建设和关心下一代工作，提升市民文明素养。

城市品质与东部地区等高对接。加强城市规划，推进“多规合一”试点和城市设计，划定历史街区。构建长江经济带综合立体交通走廊，加快机场、铁路、过江隧道施工，启动机场快速路、商合杭铁路江北枢纽、芜合高速裕溪河特大桥建设。完善以快速路网、轨道交通、停车场为重点的城市基础设施，加快地下综合管廊建设，推进通江大道城建工程，启动九华南路、长江南路、珩琅山路快速化改造。启动长江芜湖段综合整治，加快水网圩区联圩并圩，加速青弋江、裕溪河、漳河、西河治理，开工建设凤凰颈新站。建设市级智慧城市管理指挥中心，提升城市管理水平。

乡村振兴与县域发展统筹融合。推进农业供给侧结构性改革。抓好农村集体产权制度改革、“三变”改革试点、“三权分置”工作。发展农业产业化，通过清洁生产、生态循环农业等方式推动农业绿色发展。扶持乡村旅游、农村电商等新业态，培育特色小镇，建设美丽乡村。继续实施农村公路畅通工程。高质量建设城市副中心。支持无为县、南陵县按照中等城市标准加快县城建设，支持芜湖县、繁昌县按照主城区标准提升建设水平。加快农民工市民化步伐，促进农业转移人口进城落户。

（三）聚焦和谐共生，提升生态质量。

坚持节约优先。打造具有特色规模优势的绿色现代产业体系。严控“两高一资”、低水平重复建设和产能过剩项目，加快非煤矿山等领域落后产能淘汰，发展低碳循环经济。深化节约集约用地，加大土地开发复垦整理、城乡建设用地增减挂钩、工矿废弃地复垦利用力度，盘活低效用地。启动第三次全国土地调查。推进矿产资源节约集约开发利用试点。坚持地表水域“占补平衡、先补后占、等量等效”。加强生态文明宣传教育，探索环保市场化机制改革，实现生态经济化、产业生态化的绿色发展。

坚持保护优先。巩固长江干流岸线整治成果，共抓大保护，不搞大开发。推动中央和省环保督察问题整改。打好蓝天保卫战。推进重点流域水环境综合治理，“一河一策”抓好突出问题整治。规划建设龙湖湿地公园。加强城乡环境、工业园区和重点企业污染整治，建立企业环境信用记录和违法排污黑名单制度，探索建立环境行为信用评价与信贷联动机制。

坚持自然恢复为主。加强生态资源管护，把修复长江生态环境摆在压倒性位置。全面推行林长制，推进林业生态建设，提高森林覆盖率。扩大绿量，提高植绿质量，让全市域绿起来、美起来。加强水土流失综合治理，实施生态修复、植被恢复、水源涵养、河道疏浚等工程，强化重要生态功能区的强制性保护。

（四）聚焦制度体系，深化改革开放。

打好全面深化改革攻坚战。深化供给侧结构性改革。推进“放管服”改革。深化商事制度改革，推进“多证合一”、企业注册登记全程电子化，探索“证照分离”改革试点。建设安徽政务服务网芜湖分厅。深化国资国企改革，加强与央企的合作发展。支持民营企业发展。规范政府举债融资行为，防范金融风险。深化社会治理体系建设，推进失信联合惩戒和守信联合激励。深入推进全国社区治理和服务创新实验区工作。推动公共资源交易平台服务与交易系统标准化建设。

打造内陆开放新高地。围绕“优进优出”目标，提升综合保税区、出口基地等平台作用，培育出口导向型企业，鼓励新兴产业产品拓展国际市场。积极申建水果、冰鲜水产品进境指定口岸，完善跨境电子商务平台和进口商品直销中心功能。复制推广自贸试验区经验，提升通关便利化水平，推进自贸试验区申建。深化区域合作与国际合作，支持优势企业布局 “一带一路”沿线，发展对外承包工程。加快三山港对外开放。围绕“双向合作”基础，加快中德中小企业合作区、中德合作智慧产业园、中以民用安防产业园发展。优化营商环境，提升投资贸易便利化水平，引导外资投向智能制造、战略性新兴产业、现代服务业领域。

打响最佳投资地芜湖品牌。推进开发区优化整合，发挥国家级和省级开发区的平台作用，促进投资项目落地。探索建立市场化平台，保持与各类企业协会商会的紧密联系，推动徽商回归。严格落实“四督四保”制度，扎实推进项目建设。落实减税降费等降成本政策，降低市区城镇土地使用税标准。持续推进企业对接多层次资本市场，力争3家企业上市，直接融资300亿元。强化“一企一组一策”帮扶机制，推进“四送一服”双千工程，落实政银担、“税融通”、续贷过桥等政策，扩大中小企业信用贷试点，支持实体经济发展。开展第四次全国经济普查。

（五）聚焦民生福祉，推动共建共享。

打造宜居城市。投入110余亿元，实施30余项民生工程。加大市区经营性用地供应量和公租房建设分配力度，完善促进房地产市场平稳健康发展的长效机制。实施“两治三改”专项行动，治理违法建设、违法用地，改造棚户区（“城中村”）1.3万套、老旧小区43个。完善污水管网体系，新改建市区公厕143座、农贸市场33个。免除殡葬基本公共服务费用。加大社会事业投入，加快文学艺术、新闻出版、广播电视、档案、地方志等事业发展。实施中华优秀传统文化传承发展工程，力争开工建设市文化活动中心，推进国家文化消费试点城市工作，打造群众公共文化服务品牌。完善体育设施，建设生态体育公园，广泛开展全民健身活动。发展人防、气象、残疾人事业，做好民族、宗教、外事、侨务、科普等工作，支持工会、共青团、妇联等群团组织和社会组织履行职责。深化“平安芜湖”建设，健全社会公共安全立体防控、防灾减灾和应急救援体系，完善矛盾多元化解机制，全力解决信访突出问题。增强全民国家安全意识，完善国防动员体系，加强国防教育、优抚安置和双拥共建，支持驻芜部队建设。

打造宜业城市。推进精细化的人才服务机制建设，加快市、县区“一站式”服务窗口建设。完善创业服务体系，鼓励创客创新创业。建设技工强市，打造皖江人力资源公共实训基地。实施全民参保登记计划，完善城乡居民社会养老保险政策，推进工伤保险“同舟计划”。强化安全生产红线意识，完善安全生产责任体系。

打造宜游城市。深化与安徽新华发行集团、出版集团的战略合作，实施一批文化产业项目。加快城东文化旅游集聚区基础设施建设，推进旅游集散中心和芜湖古城、鸠兹古镇、松鼠小镇等项目。引导乡村旅游提档升级、集聚发展，培育乡村特色文化旅游小镇、工农业旅游示范点和研学旅游基地。

打造宜学城市。促进基础教育优质均衡，着力解决中小学生课外负担重、“大班额”等问题。实施第三期学前教育行动计划，新改建5个公办幼儿园，创建260个普惠性民办幼儿园。推进城乡义务教育一体化发展，普及高中阶段教育。提高职业教育质量，深化产教融合、校企合作，组建职教联盟。加快建设安徽工程大学国际工程师学院，筹建航空职业技术学院。办好老年教育。

打造宜养城市。深化综合医改，健全分级诊疗制度，强化重大疾病防控和妇幼保健，推进创新城市医联体试点，优化家庭医生签约服务，提升公立医院管理和技术水平，推进智慧医疗。发展中医药事业，力争开工建设中医传承与创新研究中心。建设一院儿科二期项目、三山医养结合示范园区，完成市妇幼保健院搬迁。健全食品药品监管体系。完善社区居家养老服务体系，升级改造特困人员供养机构。开展老旧小区加装电梯试点。

（六）聚焦履职担当，加强自身建设。

建设法治政府。坚持依法行政，严格落实重大执法决定法制审核制度，提升执法水平。贯彻重大事项合法性审查程序规定，推进合法性审查机制全覆盖。自觉接受人大及其常委会监督、政协民主监督和社会舆论监督。加大法治宣传和培训力度，提高运用法治思维和法治方法开展政府工作的能力。

建设服务政府。转变政府职能，深入推进“亲切服务”。完善督查考核制度，确保重大决策部署落实到位。全面提振干事创业精气神，增强服务本领，强化责任担当，严肃查处庸懒散怠行为。推进“互联网+政务服务”建设，完善“一号认证、一窗受理、一网统办、一卡通用、一端服务”的线上线下一体化服务体系。以公开为常态、不公开为例外，推进政务公开。

建设廉洁政府。开展“不忘初心、牢记使命”主题教育，贯彻落实中央八项规定和省委、市委深入推进作风建设的规定精神，树立正确政绩观，营造风清气正的良好环境。强化审计监督，坚持失责必问、问责必严，确保政令畅通。加快构建亲清新型政商关系。针对群众身边“微腐败”现象对症下药，从严查处。坚定不移惩治腐败，夺取反腐败斗争压倒性胜利。

各位代表！新的历史方位，赋予我们这代人新的历史担当。让我们紧密团结在以习近平同志为核心的党中央周围，在中共芜湖市委的坚强领导下，锐意进取，埋头苦干，不断开创芜湖跨越发展新局面，谱写人民美好生活新篇章！
